# Assessing preparation for care transition among adolescents with rheumatologic disease: a single-center assessment with patient survey

**DOI:** 10.1186/s12969-021-00544-y

**Published:** 2021-05-01

**Authors:** Jordan E. Roberts, Olha Halyabar, Carter R. Petty, Mary Beth Son

**Affiliations:** 1 Division of Immunology, Boston Children’s Hospital, Harvard Medical School, Boston, Massachusetts USA; 2 Institutional Centers for Clinical and Translational Research, Boston Children’s Hospital, Boston, Massachusetts USA

**Keywords:** Care transitions, Transition preparation, Patient education, Quality improvement

## Abstract

**Background:**

Despite the risk for poor outcomes and gaps in care in the transfer from pediatric to adult care, most pediatric rheumatology centers lack formal transition pathways. As a first step in designing a pathway, we evaluated preparation for transition in a single-center cohort of adolescents and young adults (AYA) with rheumatologic conditions using the ADolescent Assessment of Preparation for Transition (ADAPT) survey.

**Findings:**

AYA most frequently endorsed receiving counseling on taking charge of their health and remembering to take medications. Less than half reported receiving specific counseling about transferring to an adult provider.

AYA with lower education attainment compared with those who had attended some college or higher had lower scores in self-management (1.51 vs 2.52, *p* = 0.0002), prescription medication counseling (1.96 vs 2.41, *p* = 0.029), and transfer planning (0.27 vs 1.62, *p* < 0.001). AYA with a diagnosis of MCTD, Sjögren’s or SLE had higher self-management scores than those with other diagnoses (2.6 vs 1.9; *p* = 0.048). Non-white youth indicated receiving more thorough medication counseling than white youth (2.71 vs 2.07, *p* = 0.027).

When adjusting for age, educational attainment remained an independent predictor of transfer planning (*p* = 0.037). AYA with longer duration of seeing their physician had higher transition preparation scores (*p* = 0.021).

**Conclusion:**

Few AYA endorsed receiving comprehensive transition counseling, including discussion of transfer planning. Those who were younger and with lower levels of education had lower preparation scores. A long-term relationship with providers was associated with higher scores. Further research, including longitudinal assessment of transition preparation, is needed to evaluate effective processes to assist vulnerable populations.

## Introduction

The transfer from pediatric to adult care is a high-risk period for poor outcomes [1, 2]. National data show that few adolescents receive comprehensive services to prepare them adequately for the transition to adult care [3], and many adolescents and young adults (AYA) with rheumatologic conditions experience gaps in care and disease flares around the time they are transferring care [ 4, 5]. Despite these risks, most pediatric rheumatology centers lack formal pathways for transition [6], and the majority of pediatric and adult rheumatologists feel preparation for transition to adult care is inadequate [7, 8].

While there is increasing recognition of the need to develop and evaluate transition pathways in rheumatology, few studies have directly evaluated AYAs’ preparation for transition in pediatric rheumatology [9, 10]. Most recent work has assessed patient satisfaction with transition interventions [11, 12], rather than specifically evaluating the content of transition counseling delivered, or assessed rate of successful transfer [13, 14], which is a crucial metric, but may be impacted by many factors other than a transition intervention. We evaluated the content of transitional care preparation in a single pediatric rheumatology center using a validated quality measure in order to identify areas for improvement.

## Methods

AYA ages 16 and older with a confirmed rheumatologic diagnosis by ICD9 or ICD10 code, at least 3 prior visits at our rheumatology practice, and email addresses on file were identified by an electronic medical records search. Eligible AYA were emailed a version of the ADolescent Assessment of Preparation for Transition (ADAPT) survey, a validated tool developed to assess patient perception of preparation for transition in adolescents and young adults with chronic diseases [15]. At the time of this baseline survey, no written transition policy was in place and individual provider practices and counseling around the transition process were known to vary substantially. Thus, we sought to evaluate the rate of counseling on specific transition topics to identify gaps and areas for quality improvement. The ADAPT quality measure assesses transition preparation in three domains: (1) self-management, (2) prescription medications, and (3) transfer planning, using specific questions about counseling received and preparatory steps taken, such as receiving a written plan specifying transition to an adult provider. We selected the ADAPT over the TRAQ and other instruments due to its focus on patient perception of provider counseling, rather than assessing baseline transition skills. We felt this would provide the most useful information about patient education in our transition process, including giving a baseline assessment of provider counseling in our division. The survey was adapted for online completion using REDCap (Research Electronic Data Capture) [16, 17]. The tool was modified to expand the age (up to 23) and education (up to college graduate) categories, as our pediatric rheumatology program sees patients until age 22 or college graduation. AYA received an invitation email in October or November 2019, and two reminder emails were sent to non-respondents. Respondents were offered a $15 gift card for completing the survey. Responses were converted to domain composite scores (self-management, prescription medications, and transfer planning) and analyzed using descriptive statistics. A total transition composite score was computed using the percent of affirmative responses out of all transition items assessed. The association of patient illness and demographic factors with composite transition scores was performed using t-tests and analysis of variance tests. Linear and logistic regression were used to evaluate for independent predictors of transition preparation.

## Results

337 eligible AYA were identified and sent a survey invitation via email. 78 patients responded (response rate of 23%), of whom 77 had a visit within the past year and were eligible to complete the assessment tool. Mean age was 18.9 (range 16–23), 83% were female, and 86% were white. Full demographic and disease characteristics of respondents are reported in Table 1. Compared to respondents, non-respondents were 67% female, and of same age (mean 18.8 years, range 16–23). Self-identified race and ethnicity were available only for respondents. Race recorded in the electronic medical record was compared between groups and was similar, with respondents 78% white, 6% Black, 3% Asian, and 1% American Indian or Alaskan Native versus non-respondents 71% white, 7% Black, 3% Asian, and 1% American Indian or Alaskan Native. Non-respondents had a similar distribution of diagnosis to respondents, with 66% having diagnosis codes for JIA, and 17% for SLE, MCTD and/or Sjögren’s. Clinical Juvenile Arthritis Disease Activity Scores (cJADAS) were available for a visit within the past year for 65% of JIA patients who responded to the survey, with median score 2 (range 0–26), which was not significantly different from non-responders (median 1, range 0–23).

**Table 1:** Patient Characteristics

	n (%) or Mean (SD)
Race	
White	66 (86)
Black or African American	8 (10)
Asian	1 (1)
Ethnicity	
Not Hispanic, Latino, or Spanish Origin	72 (94)
Hispanic, Latino, or Spanish Origin	5 (6)
Female	64 (83)
Age (years)	18.9 (2.0)
Education	
9th grade	2 (3)
10th grade	9 (12)
11th grade	13 (17)
12th grade, high school graduate, or GED	13 (17)
Some college	33 (43)
College graduate	7 (9)
Primary Rheumatologic Diagnosis^a^	
Juvenile idiopathic arthritis	52 (68)
Uveitis (idiopathic)	2 (3)
Chronic recurrent multifocal osteomyelitis	3 (4)
Systemic lupus erythematosus	8 (10)
Mixed connective tissue disease	4 (5)
Sjögren’s	3 (4)
Juvenile dermatomyositis /inflammatory myositis	3 (4)
Other vasculitis	4 (5)
Length of time seeing doctor	
At least 6 months but less than 1 year	2 (3)
At least 1 year but less than 3 years	20 (26)
At least 3 years but less than 5 years	19 (25)
5 years or more	36 (47)
Frequency of visits in past year	
1 time	17 (22)
2 times	24 (31)
3 times	17 (22)
4 times	13 (17)
5 to 9 times	4 (5)
10 or more times	2 (3)
Health status	
Excellent	10 (13)
Very good	18 (23)
Good	32 (42)
Fair	15 (19)
Poor	2 (3)

^a^Some patients have more than 1 primary rheumatologic diagnosis

Respondents most frequently endorsed receiving counseling on taking charge of their health and remembering to take medications, but fewer than half reported receiving specific counseling about transfer to an adult provider (Table 2).

**Table 2:** Selected Transition Preparation Measures

Survey Question	Yes n (%)	No n (%)
In the last 12 months, did you talk with this provider without your parent or guardian in the room?	46 (59.7%)	31 (40.3%)
In the last 12 months, did you and this provider talk about you being more in charge of your health?	53 (68.8%)	24 (31.2%)
In the last 12 months, did you and this provider talk about whether you may need to change to a new provider who treats mostly adults?	31 (40.8%)	45 (59.2%)
In the last 12 months, did this provider ask if you had any questions or concerns about changing to a new provider who treats mostly adults?	22 (71.0%)	9 (29.0%)
In the last 12 months, did you and this provider talk about a specific plan for changing to a new provider who treats mostly adults?	14 (45.2%)	17 (54.8%)
In the last 12 months, did you and this provider talk about remembering to take your medicines?	37 (75.5%)	12 (24.5%)
Composite scores	Mean (SD)	
Self-management (*n* = 77; score out of 4)	2.0 (1.2)	
Medication counseling (*n* = 49; score out of 3)	2.2 (0.7)	
Transfer planning (*n* = 76; score out of 4)	1.0 (1.4)	
Total transition composite score (n = 77; % of items answered yes)	0.45 (0.25)	

AYA ages 16–18 had significantly lower scores in all domains compared to those 19 years and older. Those with lower education attainment compared with those who had attended some college or higher had lower scores in self-management (1.51 vs 2.52, *p* < 0.001), prescription medication counseling (1.96 vs 2.41, *p* = 0.029), and transfer planning (0.27 vs 1.62, p < 0.001). AYA with longer duration of seeing their rheumatologist had higher transition preparation scores (*p* = 0.021). When controlling for age, educational attainment remained an independent predictor of higher transfer planning scores (*p* = 0.037), but not of other measures.

AYA with a diagnosis of mixed connective tissue disease, Sjögren’s syndrome or systemic lupus erythematosus had higher self-management scores than those with other diagnoses (2.6 vs 1.9; *p* = 0.048). Non-white respondents indicated they had received more thorough medication counseling than white respondents (2.71 vs 2.07, *p* = 0.027).

Survey respondents with diagnosis of JIA (*n* = 52) reported attending 1 (23%), 2(35%), 3 (17%) or 4 (21%) rheumatology visit with their rheumatologist in the year prior to survey completion; only 4% had 5 or more visits. Those with mixed connective tissue disease, Sjögren’s syndrome or systemic lupus erythematosus (*n* = 15) reported 2 (27%), 3 (53%) or 4 (13%) visits over the same period.

## Conclusions

Despite growing awareness of the importance of the pediatric to adult care transition for AYA with chronic illnesses, few youth in our cohort endorsed receiving comprehensive transition counseling, including discussion of transfer planning. This is similar to national data, with the National Survey of Children’s Health reporting that just 18% of youth ages 12–17 received comprehensive services for care transition, defined as meeting with their pediatrician alone, addressing skill-building for transition, and receiving counseling about transfer to adult care if needed [3].

We observed demographic and disease-related differences in transition preparation in our cohort. Non-white patients and those with lupus and related conditions had significantly higher domain scores, possibly reflecting greater provider awareness of these risk factors for poor transfer outcomes, or more frequent contact with their rheumatologist.

Consistent with prior work showing increased transition readiness with older age [18–20], we found that transition preparation scores in all domains were highly correlated with age. Our study included older AYA than have typically been assessed in transition program evaluations in pediatric rheumatology, and therefore we were able to show significant gains in the 19 and up age group, which aligns with recent work in youth with inflammatory bowel disease showing that self-management skills may not be achieved until age 18 and after [21]. Therefore, it may be important to frame the young adult years as a time of continued transition skill-building, including support following a first visit with an adult provider [22].

We also found that AYA with less formal education had lower transfer planning scores even when adjusting for age. This disparity may reflect differences in self-management skills among AYA with less formal education [23]. In our program, many providers have traditionally seen young adults through their graduation from college, and therefore have used this as a milestone to counsel patients regarding transfer timelines. However, this may be less relevant for young adults not enrolled in post-secondary education, and could be a cause of lower preparedness for transfer among those with less formal education. In contrast, a longer-term relationship with a provider was associated with higher transition preparation scores.

Our study had several limitations, including a low response rate, though age, race, and diagnosis of respondents were similar to non-respondents. As this was a single-center evaluation, our results may not be generalizable to other centers with different patient populations, referral patterns, and baseline transition processes.

Following closure of the survey, our clinic began distributing a newly created written transition policy to all patients 14 and older at follow-up visits. The importance of a written transition policy has been highlighted in the Six Core Elements of Transition developed by collaborative transition workgroup of the American Academy of Pediatrics, American Academy of Family Physicians, and American College of Physicians, and promoted by the American College of Rheumatology’s Transition Toolkit [24, 25]. A written policy was selected as the first step in improving our transition process, as drafting and revising the policy afforded an opportunity for providers with diverse transition counseling practices to build consensus around priority areas for counseling and timelines for transfer, and to communicate this to families in our practice. This transition policy incorporated parent and patient input and addressed expectations for increasing patient responsibility and transition timelines. Additional initiatives were designed to distribute the policy during the COVID19 pandemic, including sending information about the transition policy via email prior to telemedicine encounters and posting the policy to the clinic’s patient information website. A repeat survey following full electronic and in-person adoption of the policy is planned to assess improvement from this intervention.

Further research including longitudinal assessment of transition preparation is needed to evaluate effective processes to improve transition outcomes. Assessment of specific transition counseling gaps using tools such as the ADAPT, and evaluation of preparation in those with different demographic characteristics and disease features may help to effectively customize transition initiatives for vulnerable patient populations.

## Data Availability

The datasets generated and analyzed during the current study are available from the corresponding author on reasonable request.

## Abbreviations

ICD: International classification of diseases
ADAPT: Adolescent assessment of preparation for transition
REDCap: Research electronic data capture
GED: General education development
